# PP22 A Dashboard To Support The Value Assessment Of Digital Health Technologies For Health Technology Assessment Agencies

**DOI:** 10.1017/S0266462324001958

**Published:** 2025-01-07

**Authors:** Gurmit Sandhu, Izzuna Mudla Mohamed Ghazali, Emanuele Laurenzi, Enkelejda Miho

## Abstract

**Introduction:**

There needs to be more awareness and agreement on value criteria important to digital health stakeholders (DHSs), including health technology assessment agencies, especially in low- and middle-income countries (LMICs). To overcome these challenges, we focused on the feasibility of a dashboard to improve low confidence and assessment readiness of digital health technologies (DHTs) among DHSs in Malaysia.

**Methods:**

“Artificial Intelligence Embedded X-ray for Lung Cancer Screening” from the Malaysian Health Technology Assessment Section (MaHTAS) was selected as a DHT use case. Representatives from MaHTAS, health informatics, research and innovation agencies, the digital health industry, patient advocates, and bioethicists attended an information briefing session and workshops. Participants completed a value assessment checklist questionnaire on the completeness of the information for the use case. Data and insights from these online workshops were incorporated into the development of Figma dashboard mock-up. Feedback from participants, other Malaysian DHSs, additional LMICs, and high-income countries was incorporated into the mock-up.

**Results:**

For the use case, average ratings from participants on the completeness of information on the seven value domains (VDs) were 3.4 and 3.1 for “healthcare system challenges and current use of technology” and “effectiveness” out of a maximum rating of 4 for “sufficient.” Both VDs were classified as “partially sufficient.” Ratings for the remaining VDs were 2.9 on “technical product information and use,” 2.8 on “safety,” 2.5 on “cost and economic impact,” 2.2 for “usability and accessibility,” and “ethical aspects.” These VDs were classified as “not sufficient.” The dashboard and its use were evaluated positively by 17 of 22 DHSs, including participants.

**Conclusions:**

It was feasible to develop a useful dashboard applying a value assessment framework for DHTs on a use case with clear visualizations and a systematic approach. Our results have potential for applications in early scientific advice. It is to be considered that workshop participants’ profiles may create a bias regarding their subjectivity in rating completeness of information on a DHT.

